# Exacerbation of Glycoprotein VI-Dependent Platelet Responses in a Rhesus Monkey Model of Type 1 Diabetes

**DOI:** 10.1155/2013/370212

**Published:** 2013-06-06

**Authors:** J. F. Arthur, Y. Shen, Y. Chen, J. Qiao, R. Ni, Y. Lu, R. K. Andrews, E. E. Gardiner, J. Cheng

**Affiliations:** ^1^Australian Centre for Blood Diseases, Alfred Medical Research & Education Precinct (AMREP), Monash University, Melbourne, VIC 3004, Australia; ^2^Key Laboratory of Transplant Engineering and Immunology, West China Hospital, Ministry of Health, Sichuan University, Chengdu 610041, China

## Abstract

Thrombosis is a life-threatening complication of diabetes. Platelet reactivity is crucial to thrombus formation, particularly in arterial vessels and in thrombotic complications causing myocardial infarction or ischaemic stroke, but diabetic patients often respond poorly to current antiplatelet medication. In this study, we used a nonhuman primate model of Type 1 diabetes to measure early downstream signalling events following engagement of the major platelet collagen receptor, glycoprotein (GP)VI. Diabetic monkeys were given enough insulin to maintain their blood glucose levels either at ~8 mM (well-controlled diabetes) or ~15 mM (poorly controlled diabetes). Flow cytometric analysis was used to measure platelet reactive oxygen species (ROS) generation, calcium mobilisation, receptor surface expression, and immature platelet fraction. We observed exacerbated intracellular ROS and calcium flux associated with engagement of GPVI in monkeys with poorly controlled diabetes. GPVI surface levels did not differ between healthy monkeys or the two diabetic groups. Treatment of platelets with the specific Syk inhibitor BAY61-3606 inhibited GPVI-dependent ROS and, importantly, reduced ROS generation in the poorly controlled diabetes group to that observed in healthy monkeys. These data indicate that glycaemic control is important in reducing GPVI-dependent platelet hyperreactivity and point to a potential antithrombotic therapeutic benefit of Syk inhibition in hyperglycaemic diabetes.

## 1. Introduction

Type 2 diabetes occurs when individuals with an underlying genetic disposition develop resistance to the glucose uptake/metabolism-promoting signals of insulin. Type 1 diabetes, which accounts for 5%–10% of all diabetes cases, generally results from autoimmune destruction of insulin-secreting pancreatic islet cells [1]. One of the high risk complications of diabetes is thrombosis, and platelets are pivotal to thrombus formation, particularly in arterial vessels, and hence the resultant thrombotic complications of myocardial infarction or ischaemic stroke. Platelets from individuals with diabetes, particularly Type 2 diabetes, are more sensitive to aggregation by a variety of agonists [2, 3], and 80% of diabetic patients are likely to die from thrombotic complications [4]. Considerably less is known about platelet activation in Type 1 diabetes, although the relative risk of cardiovascular disease in Type 1 diabetic patients can be as much as 10-fold greater than that in nondiabetic individuals [1]. However, the reduction of thrombotic events in diabetic patients has proven to be only marginal in several trials of antiplatelet therapy [5–7], and diabetes is consistently associated with a high rate of adverse cardiovascular events. There is therefore a pressing need for alternative/improved antiplatelet therapy for diabetic patients.

Exogenous reactive oxygen species (ROS) can influence platelet function, and platelets themselves are able to generate ROS. We have shown that the major collagen receptor on platelets, glycoprotein (GP) VI, is linked to redox signalling pathways via its association with the adaptor molecule, tumour necrosis factor receptor-associated factor 4 (TRAF4; [8]). TRAF4 interacts with an intracellular sequence of GPVI as well as p47^phox^ of the NADPH oxidase complex, the major source of ROS in platelets. Initiation of GPVI-dependent signalling involves GPVI-associated Lyn phosphorylating an immunoreceptor tyrosine-based activation motif (ITAM) in FcR*γ* (in a complex with GPVI), leading to recruitment and activation of spleen tyrosine kinase (Syk). A subsequent tyrosine phosphorylation signalling cascade results in intraplatelet calcium mobilisation and activation of the integrin *α*_IIb_ *β*_3_, leading to platelet aggregation and metalloproteinase-dependent GPVI ectodomain shedding. Intracellular ROS generation following GPVI engagement is an early signalling event and precedes GPVI-dependent signalling leading to aggregation and shedding. We have recently shown that GPVI ROS generation is comprised of two phases: an initial Syk-independent burst followed by additional Syk-dependent generation [9]. In the current study, we investigate GPVI-dependent ROS generation and calcium mobilisation in a rhesus model of Type 1 diabetes. Compared with rodent models, monkeys are metabolically closer to humans, and a longer lifespan allows long-term disease progression to be assessed in the same animal over 3–5 years. Genetic variability in monkeys also better approximates disease in humans compared with in-bred rodents. Importantly, while the GPVI primary sequence and platelet immunoreceptor signalling pathways are significantly different in rats or mice compared with human, monkey GPVI is closely related [10] making them suitable for platelet functional analysis. Additionally, we obtain data on GPVI-dependent ROS generation in monkeys in the setting of diabetes with controlled dietary intake to eliminate this unavoidable variable associated with human studies. We assess the influence of glycaemic control (well-controlled versus poorly controlled) in this model and the effect of Syk inhibition on ROS generation in the different diabetic groups.

## 2. Materials and Methods

### 2.1. Materials

The GPVI-specific agonist, collagen-related peptide (CRP) was prepared as previously described [11, 12]. Thrombin receptor-agonist peptide (TRAP) was from Auspep (Melbourne, VIC, Australia). The potent and selective Syk inhibitor, 2-[[7-(3,4-dimethoxyphenyl)imidazo[1,2-c]pyrimidin-5-yl]amino]pyridine-3-carboxamide hydrochloride (BAY61-3606) was from Enzo Life Sciences (Farmingdale, NY, USA).

### 2.2. Animals

Eighteen rhesus monkeys (aged 4–9) were obtained from Chengdu Ping'an Experimental Animal Reproduction Center (Sichuan, China). All the animals had free access to water supply and were fed a primate diet twice a day. The animals were cared for in accordance with the guidelines of the Experimental Animal Center, Sichuan University, which have been approved by the Association for the Assessment and Accreditation of Laboratory Animal Care International (AAALAC). Each subject had a history, recording the general condition (body weight, food intake, and behavioural activity), a physical examination, a mental health evaluation, and laboratory testing every 1-2 months. 

### 2.3. Induction of Diabetes

Monkeys were made to fast overnight. Streptozotocin (STZ; Yuyang High-tech Development Co. Ltd., Chengdu, China) was administered by intravenous injection after dissolution with sodium citrate (pH 4.5) at a dose of 80 mg/kg over 30 seconds (see [13]). Blood glucose levels were determined twice a day commencing 72 hours after STZ induction. The model was considered to be successfully established if the fasting blood glucose (FBG) values remained >11.1 mM for two consecutive days and the C-peptide concentration was <0.17 nM. After the successful induction of diabetes, monkeys were administered porcine insulin (Wanbang Biopharma Co. Ltd., Xuzhou, China) that included a combination of protamine zinc insulin and insulin, twice a day before feeding (9:00 AM and 4:30 PM) [14]. Diabetic monkeys were separated into two groups: treated with sufficient insulin to maintain the FBG level at <10 mM (well-controlled group, *n* = 6) and reduced insulin to maintain the FBG level at 15–20 mM (poorly controlled diabetes group, *n* = 6). Six healthy monkeys were used as normal controls. Experiments were conducted between July and August 2011. Diabetes was successfully established in the poorly controlled diabetic monkeys between 2 and 3 years prior to experimentation (July 2008–July 2009). Diabetes was established in the well-controlled diabetic monkeys 1 year prior to experimentation (June 2010).

### 2.4. Blood Collection

Platelet-rich plasma (PRP) was obtained from blood collected into 3.2% (w/v) trisodium citrate and centrifuged at 110 g for 20 min at room temperature. 

### 2.5. Measurement of Intracellular ROS

 Platelets in PRP from healthy, low glucose diabetic, and high glucose diabetic rhesus monkeys were loaded with 2′,7′-dichlorofluorescein (H_2_DCF-DA; Sigma, St. Louis, MO, USA), a cell-permeable nonfluorescent dye that is cleaved by intracellular esterases to H_2_DCF rendering it membrane-impermeable and then emits fluorescent energy in the presence of ROS [15–17]. H_2_DCF-DA (10 *μ*M final concentration) was incubated with PRP for 30 min at 37°C. Samples were then treated with (final concentrations) 10 *μ*g/mL CRP or 5 *μ*M TRAP, then diluted tenfold in Ca^2+^-free Tyrode's buffer (0.36 mM NaH_2_PO_4_, 5 mM HEPES, 137 mM NaCl, 5.6 mM glucose, and 2.7 mM KCl, pH 7.4) containing 0.1% (w/v) BSA and 10 *μ*M H_2_DCF-DA, and analysed in a Beckman Elite ESP flow cytometer (Beckman Coulter). Experiments were carried out in the presence or absence of Syk inhibitor (pretreatment with 5 *μ*M BAY61-3606 for 15 min) to represent the initial and secondary phases of ROS, respectively [9].

### 2.6. Measurement of Intracellular Calcium Mobilisation

The method was modified for platelet-rich plasma (PRP) from previous studies [18, 19]. In brief, Fluo-3-AM- (fluo-3 acetoxymethyl ester) labelled PRP containing 1-2 × 10^6^ platelets/mL was diluted in 290 *μ*L HEPES-Tyrode's buffer (see measurement of intracellular ROS) containing 1 mM CaCl_2_. The GPVI agonist CRP (10 *μ*g/mL) was added to the platelet/buffer sample. Platelet intracellular calcium mobilisation was measured from 10 seconds (initial calcium mobilisation) to 2.5 minutes of agonist treatment (maximal calcium mobilisation). Platelets were specifically gated on forward and side scatter characteristics and CD41a positive staining. The Fluo-3 intensity log ratio of the agonist-induced Fluo-3 positive platelets to basal (resting) level Fluo-3-labelled platelets was used for calcium mobilisation analysis. 

### 2.7. Analysis of Receptor Surface Expression

Platelet surface levels of GPVI were assessed using anti-GPVI mAb 1G5 [20, 21] directly conjugated to phycoerythrin (PE) using the Lightning-Link R-PE antibody labelling kit (Novus Biologicals, CO, USA). GPIb*α* levels were measured using PE-conjugated AK2 [22]. Integrin *α*_IIb_ *β*_3_ and platelet tetraspanin CD9 levels were measured by PE-conjugated anti-CD41a against integrin chain *α*_IIb_ and anti-CD9 mAb (PE-CD9), both from BD Biosciences, San Jose, CA, USA.

### 2.8. Measurement of Immature Platelet Fraction

Immature platelets recently released from the bone marrow contain more RNA than platelets that have been in the circulation for longer periods of time (average platelet lifespan 7–10 days). The immature platelet fraction is determined by staining platelets with the RNA dye thiazole orange (TO) and reflects platelet production and the rate of platelet turnover [23]. Platelets in PRP were fixed with 1% (w/v) paraformaldehyde for 10 min at RT. TO (0.5 *μ*g/mL final concentration in PBS containing 5 mM EDTA) was added to the fixed sample, incubated in the dark for 1 h at RT, and then analysed by flow cytometry on the FL-1 channel.

### 2.9. Statistical Analyses

 For comparison of ROS generation by various treatments, data were assessed by linear mixed model using SAS System Software Version 9.2 (SAS Institute, Cary, NC, USA). For comparison of basal ROS, peak calcium mobilisation, body weight, platelet count, mean platelet volume (MPV), platelet distribution width (PDW), and receptor levels, data were assessed by one-way ANOVA with Newman-Keuls multiple comparison post hoc test using GraphPad Prism 5.

## 3. Results

### 3.1. Haematological Parameters

**Figure 1:**
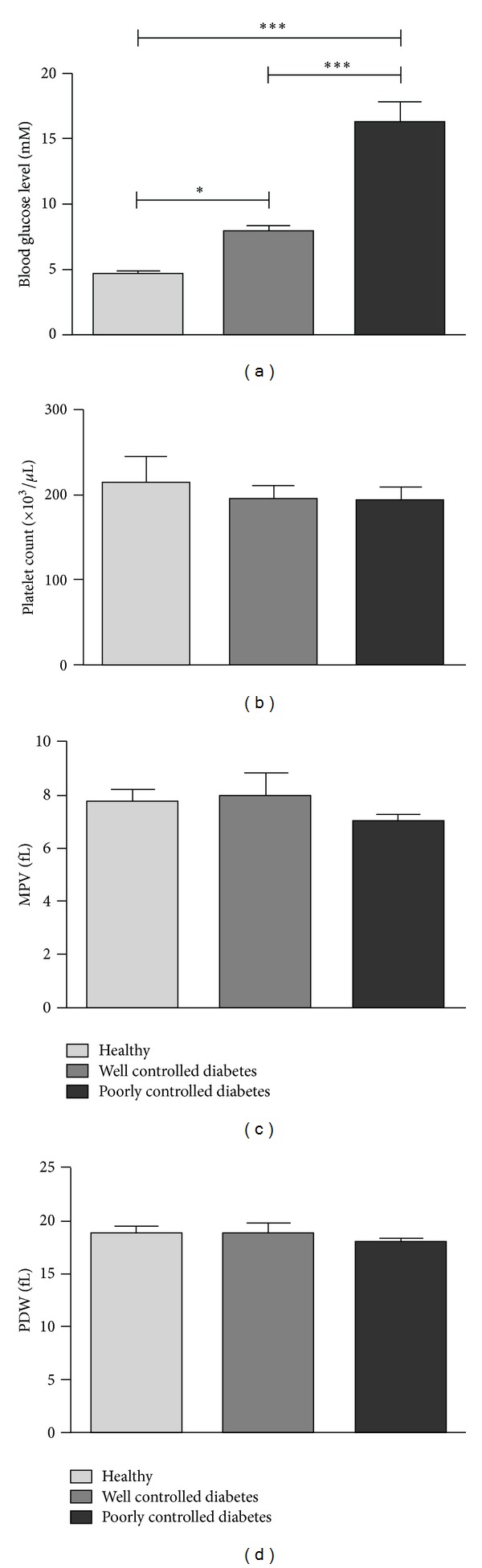
Haematological parameters for healthy and diabetic monkeys. (a) Blood glucose, (b) platelet count, (c) mean platelet volume, and (d) platelet distribution width. Data are from six monkeys in each group. *P < 0.05, ***P < 0.001.

Monkeys receiving sufficient insulin per day to control their blood glucose level (well-controlled diabetes) still had elevated glucose compared with healthy monkeys; however the blood glucose level of monkeys receiving a reduced insulin regime (poorly controlled diabetes) was significantly greater than healthy monkeys or monkeys with well-controlled diabetes (Figure 1(a)). Platelet count (Figure 1(b)) and platelet morphology markers (including mean platelet volume, Figure 1(c) and platelet distribution width, Figure 1(d)) displayed no significant difference between all three groups. Although well-controlled diabetic animals had lower average body weights than poorly controlled diabetic animals (5.7 ± 0.5 and 8.5 ± 2.1 kg for well-controlled and poorly controlled diabetic monkeys, resp.; *P* < 0.05), there was no significant difference between the average weight of healthy monkeys (7.3 ± 2.1 kg) and either diabetes group. Similarly, there was no significant difference between the mean age within each group (Table 1).

### 3.2. Intracellular ROS Generation

**Figure 2:**
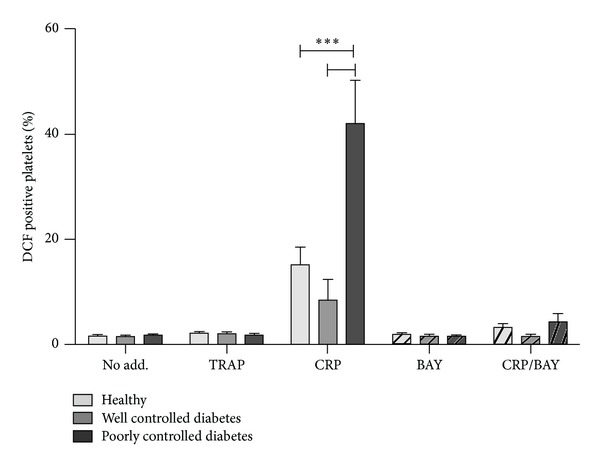
Effect of well-controlled or poor glycaemic control on intracellular ROS measurements in diabetes. Flow cytometry of H_2_DCF-DA-loaded monkey platelets in PRP either untreated (no add.) or treated with 10 *μ*g/mL CRP or 5 *μ*M TRAP. Syk inhibition by 5 *μ*M BAY61-3606 (BAY) reduces GPVI-induced ROS generation in all monkey groups. Data are from six monkeys in each group. ***P < 0.001.

To evaluate the effect of glucose control on early GPVI-mediated ROS generation in diabetic monkeys, we utilised a flow cytometry-based assay to rapidly assess intracellular ROS using the fluorescent dye, H_2_DCF-DA. Basal ROS in platelet-rich plasma (PRP) from all three monkey groups was similar (Table 1). Treatment of platelets with 10 *μ*g/mL of the GPVI-specific agonist collagen-related peptide (CRP) for 2 min increased intraplatelet ROS in all groups, but there was a significant exacerbation of CRP-induced ROS generation in the poorly controlled diabetic monkeys (Figure 2). There was a tendency for intracellular ROS generation in the well-controlled diabetic monkeys to be lower compared with healthy or poorly controlled diabetic monkeys, but this did not achieve statistical significance (*P* = 0.094). As seen previously in healthy human donors, there was no intracellular ROS generated by treatment of platelets with the thrombin receptor agonist, TRAP (5 *μ*M).

To examine early GPVI signalling events, platelets were treated with GPVI agonist in the presence and absence of 5 *μ*M BAY61-3606, a potent, specific inhibitor of Syk [24]. Increased DCF fluorescence in response to CRP occurs in two distinct phases: an initial burst of ROS occurring within 2 min followed by additional ROS generation as a consequence of downstream signalling [9]. Syk inhibition reduced CRP-induced ROS in all monkey groups and, critically, reduced the exacerbated ROS in poorly controlled diabetic monkeys to levels observed in healthy monkeys (Figure 2).

### 3.3. Intracellular Calcium Flux

**Figure 3:**
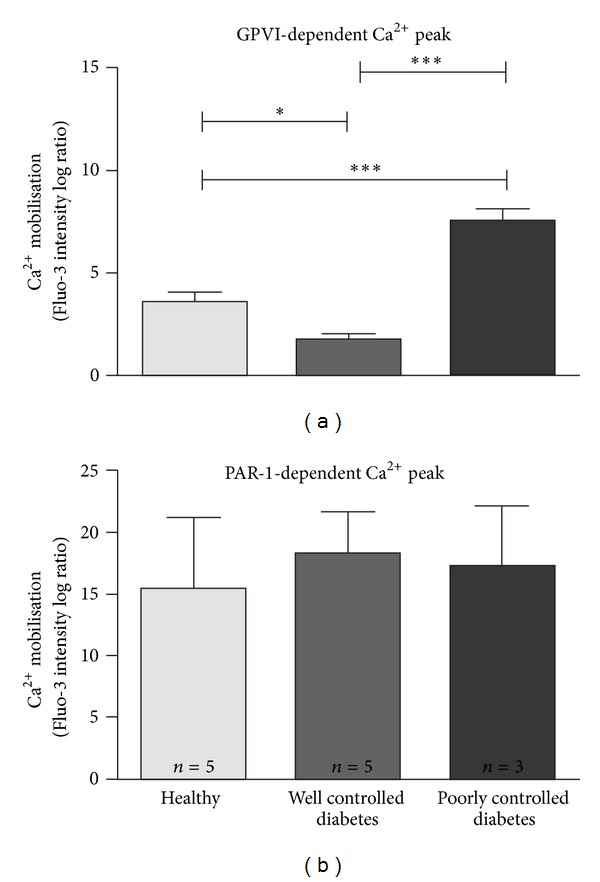
Influence of well- and poorly controlled diabetes on intracellular calcium mobilisation. Flow cytometry of Fluo-3-AM-loaded monkey platelets in PRP treated with 10 *μ*g/mL CRP (a) or 10 *μ*M TRAP (b). Data are from three to five monkeys in each group. *P < 0.05, ***P < 0.001.

As an additional readout of downstream signalling following agonist treatment of platelets, intracellular calcium mobilisation was measured by flow cytometry following treatment of platelets with 10 *μ*g/mL CRP or 10 *μ*M TRAP. Although measurements were unable to be obtained in all monkeys, calcium flux was measured in five healthy, five well-controlled diabetic and three poorly controlled diabetic animals (Figure 3). Mirroring the results obtained for intracellular ROS, peak intracellular calcium, measured 30 sec after CRP treatment, was significantly increased in poorly controlled diabetic monkeys (Figure 3(a); *P* < 0.001 compared with healthy or well-controlled diabetes). Control of blood glucose levels in diabetic monkeys reduced the peak calcium flux to below that observed in healthy monkeys (*P* < 0.05). In contrast, peak calcium flux induced by the PAR-1 agonist TRAP (measured at 10 sec after treatment) was equivalent in all monkey groups (Figure 3(b)). 

### 3.4. Receptor Surface Expression

**Figure 4:**
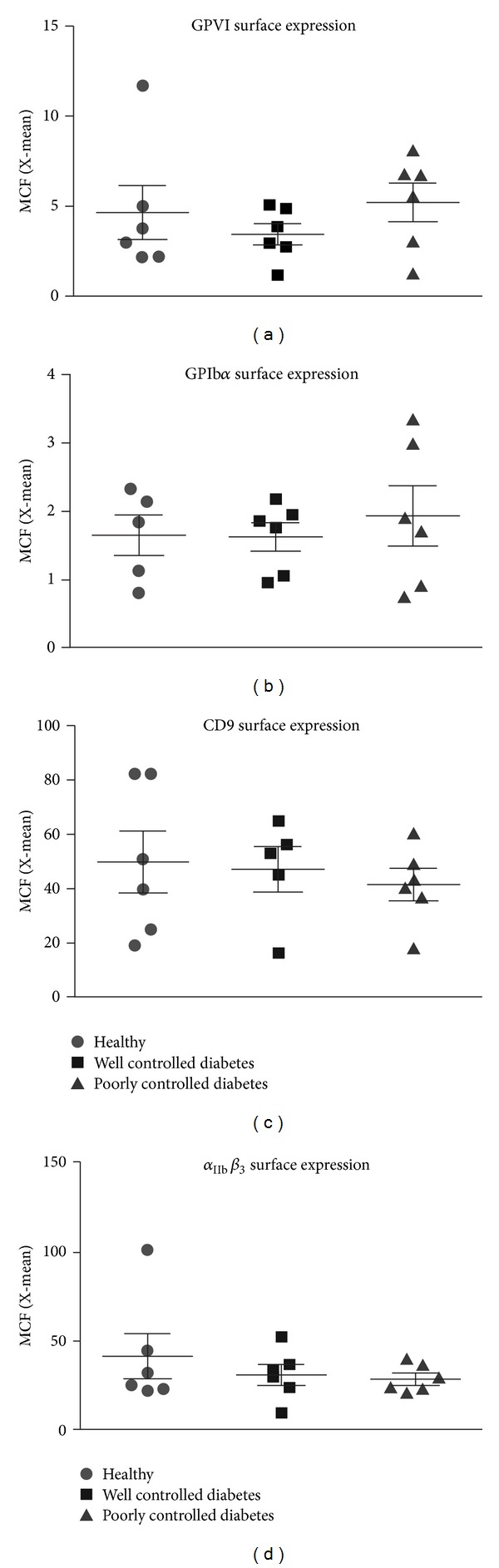
Effect of diabetes on platelet receptor surface levels. Platelet surface expression of (a) GPVI, (b) GPIb*α*, (c) CD9, and (d) *α*_IIb_ *β*_3_. Data are from five to six monkeys in each group.

 Functional GPVI responses are determined at least in part by surface density of GPVI [25, 26], so to assess whether variation in surface expression could account for differences in intracellular ROS production or calcium flux observed between the diabetes groups, receptor surface levels of GPVI and three other platelet receptors were measured. Expression of glycoprotein receptors (GPVI, GPIb*α*) using in-house PE-labelled antibodies displayed lower mean fluorescence intensity than that of the corresponding measurements of CD9 (anti-tetraspanin antibody) or *α*_IIb_ *β*_3_ using commercial PE-labelled antibodies. GPVI surface expression was similar between all three monkey groups: healthy, well-controlled diabetes, and poorly controlled diabetes (Figure 4(a)). Although an outlier in the healthy group (confirmed by extreme studentized deviate method (ESD, Grubbs' test, GraphPad)) raised the average expression level of this group, exclusion of the outlier did not alter the overall outcome but did highlight the tendency for GPVI expression in the poorly controlled diabetic animals to be higher than in the other two groups. There was no difference in surface levels of GPIb*α*, CD9, or *α*_IIb_ *β*_3_ between groups (Figures 4(b)–4(d)). TO staining of platelets to detect the immature platelet fraction similarly revealed no differences (Table 1) suggesting that platelet hyperactivity was not likely to be due to aberrant platelet production or clearance. 

## 4. Discussion

Type 1 diabetic patients have increased risk of cardiovascular disease compared with non-diabetic individuals [1]. Platelet hyperreactivity plays a key role in prothrombotic complications. Increased platelet responsiveness to collagen has been proposed as a contributing factor to the increased incidence of vascular disease seen in diabetes. In a non-human primate model of Type 1 diabetes, we observed exacerbated intracellular ROS and calcium flux associated with engagement of GPVI, the major platelet collagen receptor on platelets. 

 Basal levels of intraplatelet ROS were not different between healthy monkeys and monkeys receiving sufficient insulin to control their diabetes (well-controlled) or those receiving less insulin (poorly controlled). This was not the case in a study of non-insulin-dependent diabetic patients in whom resting platelets had a basal level of ROS that was significantly higher than in controls [27]. The patients in that study had an average age of 60 years so it is likely that their platelets had been subjected to longer periods of hyperglycaemia than our monkeys. It will be interesting to perform follow-up studies in our monkeys to determine whether the oxidative status of their platelets is altered with time.

In diabetic monkeys with higher levels of blood glucose, there was a significant increase in GPVI-dependent ROS generation compared with normoglycaemic monkeys or diabetic monkeys with lower levels of blood glucose. Hyperglycaemia is the diagnostic feature of Type 1 and Type 2 diabetes, yet the role of glycaemic control in preventing thrombotic events is not easily defined. Randomised controlled trials involving Type 1 and Type 2 diabetic patients have found that improved glycaemic control might stabilise macrovascular disease or prevent progression in those at risk [28]. In Type 1 diabetic patients with established microvascular complications of nephropathy, however, a significant improvement in glycaemic control did not improve hyperreactivity of platelets* in vitro* [29]. In a study involving Type 2 diabetic patients, Davi and colleagues [30] found that only with tight glycaemic control there was resultant improvement in platelet function. Our data indicate that specific hyperreactivity to GPVI engagement is an early event in the progression of Type 1 diabetes and is linked to high blood glucose levels. This is in agreement with a recent study in which moderately controlled diabetes (~10–13 mM blood glucose) was induced in cynomolgus monkeys fed an atherogenic diet, and an increase in ROS was detected prior to atherosclerotic changes [31], supporting ROS elevation as an initiating event in diabetic vascular disease.

Of interest, monkeys with well-controlled diabetes in our study had reduced GPVI-dependent platelet responses relative to the healthy controls, despite having elevated glucose levels. The reduction was statistically significant for calcium responses but did not achieve significance for the ROS measurements. Insulin has been reported to have antiplatelet effects, inhibiting platelet interaction with collagen-coated coverslips following *in vivo* perfusion in nonobese, non-diabetic individuals and reducing aggregation induced by several agonists [32]. Although there is still considerable debate over whether insulin has a direct effect on platelet function [33, 34], our data could point to a role for insulin administration specifically reducing GPVI-dependent signalling (ROS generation and calcium mobilisation). Although it is possible that monkey characteristics in the well-controlled diabetes group (i.e., more females, lower average weight and age) could have contributed to the observed reduced platelet responsiveness, it was not a global platelet phenomenon because signalling downstream of G protein-coupled PAR-1 receptors was not diminished under the same conditions (Figure 3). 

Mean platelet volume (MPV) is probably the most extensively studied morphological marker of platelet activation. Platelet distribution width (PDW) indicates the variation in platelet size and is also used as an indication of platelet activation, with both increased MPV and higher PDW associated with increased platelet activation. In a recent study evaluating platelet indices in diabetic and non-diabetic patients, MPV and PDW were significantly higher in diabetic patients compared to the control subjects [35]. Among the diabetic patients, PDW was found to be higher in those with microvascular complications (retinopathy, nephropathy, and neuropathy) compared to those without. In our monkey model, there was no difference in either MPV or PDW between healthy animals or the two diabetic groups. In accordance with these observations, there was also no difference in TO staining (a measure of the immature platelet fraction which usually comprises larger, more haemostatically active platelets) between the different monkey cohorts indicating that there was no difference in platelet turnover. Diabetic patients have been reported to have accelerated platelet consumption/production and hence an increase in immature platelets [36]. Given that our diabetic models have been established no longer than three years (the earliest established was a poorly controlled diabetic monkey, in July 2008, 3 years prior to our laboratory tests), the increase in MPV, PDW, and immature platelets associated with diabetes with/without vascular problems could be a later development of the disease. 

Surface expression of FcR*γ*/GPVI is reportedly enhanced on platelets of Type 2 diabetic individuals, although only FcR*γ* chain levels correlated significantly with diabetes [37]. No data has been obtained in Type 1 diabetic patients. In the current study, there was a tendency for monkeys with poorly controlled diabetes to have higher levels of GPVI on the platelet surface; however this did not reach statistical significance. The reason for exacerbated GPVI-dependent signalling in the hyperglycaemic monkeys was therefore unlikely to be due to a higher density of receptors. There was no difference in expression of other platelet receptors, GPIb*α*, CD9, or integrin *α*_IIb_ *β*_3_, between the groups.

Enhanced calcium mobilisation has been observed in platelets from Type 1 and Type 2 diabetic patients [17, 38]. Intracellular calcium homeostasis in platelets of patients with non-insulin-dependent diabetes is altered, leading to increased adhesiveness and spontaneous aggregation [39]. In our poorly controlled Type 1 diabetic monkeys, peak calcium mobilisation was significantly increased relative to healthy or well-controlled diabetic monkeys following treatment with the GPVI-specific ligand, CRP. No such hyperreactivity was observed when platelets were treated with the PAR-1 agonist, TRAP, indicating that the exaggerated calcium response is specific for GPVI engagement at this time (≤3 years) of disease progression. 

Upon GPVI engagement, phosphorylation of the ITAM motif of the associated FcR*γ* chain initiates a tyrosine phosphorylation-dependent signalling cascade that involves Syk activation as an early event and leads to platelet activation. Unlike the initial burst of ROS production following GPVI engagement, the secondary phase is completely blocked by the Syk inhibitor, BAY 61-3606, which inhibits GPVI-dependent signalling leading to activation of *α*_IIb_ *β*_3_ [9]. Inhibition of Syk in the current study reduced CRP-induced ROS generation regardless of the level of glycaemic control. Early ROS generation in poorly controlled diabetic animals was indistinguishable from that in healthy animals. Basal ROS levels were similar in all groups so this indicates that the hyperreactivity associated with hyperglycaemia in this model of diabetes was due to increased signalling downstream of Syk rather than an upregulation of receptor proximal signalling. Syk inhibition could prove to be an effective antiplatelet strategy for diabetic patients because of its minimal impact on primary haemostasis while providing significant protection from arterial thrombosis [40]. The potential for targeting Syk in autoimmune diabetes has been evaluated in a recent mouse study. Oral administration of the Syk inhibitor R788 delayed the onset of spontaneous diabetes in nonobese diabetic (NOD) mice and the progression of early-established diabetes even when treatment was initiated after the development of glucose intolerance in those animals [41]. The mechanism in that study was reportedly blockage of B cell receptor- and Fc*γ*R-mediated antigen presentation due to Syk inhibition although R788 treatment was not associated with large reductions in autoantibody levels, suggesting that perhaps a reduction in B cell development and activation was not the main reason for the antidiabetic effect of Syk inhibition.

Mechanistically, what could be the link between GPVI and hyperglycaemia? Aldose reductase (AR) is the first enzyme of the polyol pathway, which converts excess glucose to sorbitol accompanied by an increase in the cytosolic NADH/NAD+ ratio. Under normal glycaemic conditions, AR is only a minor consumer of glucose; however during hyperglycaemia AR activity is significantly enhanced and it is thought to contribute to the vascular complications associated with diabetes by increasing oxidative and osmotic stress on cells. Two important recent studies have linked AR and GPVI signalling [42, 43]. First, proteomic analysis of differentially altered proteins revealed that AR activity and expression were upregulated following GPVI-dependent platelet activation [42]. These changes were functionally relevant because inhibition of AR activity resulted in reduced GPVI-dependent platelet aggregation. Second, AR was shown to play a central role in GPVI-dependent signal transduction (increased PLC*γ*2 and PKC activation) and this signalling pathway was enhanced in hyperglycaemic conditions [43]. What is not known is how AR fits into the GPVI signalling cascade and how glucose metabolism through AR might enhance GPVI-dependent platelet responses. Further investigation is required and is the subject of an ongoing study.

In conclusion, we have demonstrated exacerbated platelet intracellular ROS and calcium flux associated with engagement of GPVI in monkeys with poorly controlled diabetes and that inhibition of a specific signalling protein (Syk) reduced GPVI-dependent ROS generation regardless of the level of glycaemic control in diabetic monkeys, indicating that Syk inhibition could prove to be an effective antiplatelet strategy for diabetic patients.

## Figures and Tables

**Table 1:** Comparison of monkey parameters.

	Healthy	Well-controlled diabetes	Poorly-controlled diabetes
*n*	6	6	6
Age (years)^†^	5.50 ± 0.96 (4–9)	4.17 ± 0.17 (4–5)	5.67 ± 0.21 (5–6)
Recent weight (kg) (July-Aug. 2011)	7.25 ± 2.06	5.70 ± 0.54*	8.47 ± 2.09
Gender	4 M, 2 F	2 M, 4 F	6 M
Monthly blood glucose level (mM)^†^ (July-Aug. 2011)	4.7 ± 0. 15 (4.2–5.2)	7.87 ± 0.53* (2.6–18.20)	16.17 ± 1.65*** (8.0–31.2)
Platelet count in whole blood^†^	213.5 ± 31.5 (139–330)	192.8 ± 17.5 (137–241) *n* = 5	193.4 ± 15.3 (150–230) *n* = 5
MPV^†^	7.78 ± 0.86 (7.3–9.31) *n* = 5	7.98 ± 1.67 (7.07–10.7) *n* = 5	7.01 ± 0.54 (6.56–7.95) *n* = 5
Basal ROS (X-mean)	0.82 ± 0.05	0.80 ± 0.06	0.85 ± 0.05
Immature platelet fraction (TO staining; X-mean)	30.8 ± 3.6	29.8 ± 3.7	27.4 ± 3.9

^†^Mean ± standard error (range).

*P < 0.05; ***P < 0.001 compared with control.

## Abbreviations

CRP: Collagen-related peptide
FBG: Fasting blood glucose
GP: Glycoprotein
H_2_DCF-DA: 2′,7′-dichlorodihydrofluorescein diacetate
ITAM: Immunoreceptor tyrosine-based activation motif
MPV: Mean platelet volume
PAR-1: Protease activated receptor-1
PDW: Platelet distribution width
PE: Phycoerythrin
PRP: Platelet-rich plasma
ROS: Reactive oxygen species
STZ: Streptozotocin
Syk: Spleen tyrosine kinase
TO: Thiazole orange
TRAF4: Tumour necrosis factor receptor-associated factor 4
TRAP: Thrombin receptor-activating peptide.
